# Supplementary material for: On the Accessibility of Adaptive Phenotypes of a Bacterial Metabolic Network
Source: PLoS Comput Biol. 2009 Aug 21;5(8):e1000472. doi: 10.1371/journal.pcbi.1000472 (PMC2716542; doi:10.1371/journal.pcbi.1000472)
Supplement: Table S1 — Reactions found in the E. coli central metabolic network analyzed in this study (0.14 MB DOC) [file pcbi.1000472.s001.doc]

**Table S1. Reactions found in the *E. coli* central metabolic network analyzed in this study**

| **Reaction** | **Protein/ protein complex** | **ORFs** | **Description** |
| --- | --- | --- | --- |
| ACCOA + OA --> COA + CIT | GltA | b0720 | Citric acid cycle |
| CIT --> OA + AC | CitDEF | b0615,b0616,b0617 |
| MAL --> OA | Mqo | b2210 |
| CIT <--> ICIT | AcnA | b1276 |
| CIT <--> ICIT | AcnB | b0118 |
| ICIT + NADP <--> CO2 + NADPH + AKG | Icd | b1136 |
| AKG + NAD + COA --> CO2 + NADH + SUCCOA | LpdA and SucAec and SucBec | b0726,b0727,b0116 |
| SUCCOA + ADP + PI <--> ATP + COA + SUCC | Frd | b0728,b0729 |
| FUM <--> MAL | FumA | b1612 |
| FUM <--> MAL | FumB | b4122 |
| FUM <--> MAL | FumC | b1611 |
| MAL + NAD --> NADH + OA | Mdh | b3236 |
| GLC + ATP --> G6P + ADP | Glk | b2388 | Glycolysis/ gluconeogenesis |
| G6P <--> F6P | Pgi | b4025 |
| F6P + ATP --> FDP + ADP | PfkA | b3916 |
| F6P + ATP --> FDP + ADP | PfkB | b1723 |
| FDP --> F6P + PI | Fbp | b4232 |
| FDP <--> T3P1 + T3P2 | FbaA | b2925 |
| FDP <--> T3P1 + T3P2 | FbaB | b2097 |
| FDP <--> T3P1 + T3P2 | B1773 | b1773 |
| T3P2 <--> T3P1 | Tpi | b3919 |
| T3P1 + PI + NAD <--> NADH + 13PDG | GapA | b1779 |
| FDP --> F6P + PI | GlpX | b3925 |
| 13PDG + ADP <--> 3PG + ATP | Pgk | b2926 |
| 3PG <--> 2PG | GpmB | b4395 |
| 3PG <--> 2PG | GpmA | b0755 |
| 3PG <--> 2PG | YibO | b3612 |
| 2PG <--> PEP | Eno | b2779 |
| PYR + ATP --> PEP + AMP + PI | Ppsa | b1702 |
| PEP + ADP --> PYR + ATP | Pykf | b1676 |
| PEP + ADP --> PYR + ATP | Pyka | b1854 |
| PYR + COA + NAD --> NADH + CO2 + ACCOA | AceEec and AceFec and LpdA | b0114,b0115,b0116 |
| G6P + NADP <--> D6PGL + NADPH | Zwf | b1852 | Pentose phosphate pathway |
| D6PGL --> D6PGC | PGL | b0767 |
| D6PGC + NADP --> NADPH + CO2 + RL5P | Gnd | b2029 |
| RL5P <--> R5P | RpiA | b2914 |
| RL5P <--> R5P | RpiB | b4090 |
| RL5P <--> X5P | Rpeec | b3386 |
| RL5P <--> X5P | SgcE | b4301 |
| R5P + X5P <--> T3P1 + S7P | TktA | b2935 |
| X5P + E4P <--> F6P + T3P1 | TktB | b2465 |
| T3P1 + S7P <--> E4P + F6P | TalB | b0008 |
| R5P + X5P <--> T3P1 + S7P | TalA | b2465 |
| X5P + E4P <--> F6P + T3P1 | TktA | b2935 |
| T3P1 + S7P <--> E4P + F6P | TktB | b2464 |
| OA --> CO2 + PYR | Eda | b1850 |
| ACCOA + 2 NADH <--> ETH + 2 NAD + COA | AdhE | b1241 | Pyruvate metabolism |
| PYR + COA --> ACCOA + FOR | PflC | b3951,b3952 |
| PYR + COA --> ACCOA + FOR | TdcE | b3114 |
| PYR + COA --> ACCOA + FOR | PflA | b0902,b0903 |
| ACCOA + PI <--> ACTP + COA | Pta | b2297 |
| ACCOA + PI <--> ACTP + COA | EutD | b2458 |
| ACTP + ADP <--> ATP + AC | AckA | b2296 |
| ACTP + ADP <--> ATP + AC | PurT | b1849 |
| ACTP + ADP <--> ATP + AC | TdcD | b3115 |
| ATP + AC + COA --> AMP + PPI + ACCOA | Acs | b4069 |
| OA + ATP --> PEP + CO2 + ADP | Pck | b3403 | Anaplerotic reactions |
| PEP + CO2 --> OA + PI | Ppc | b3956 |
| MAL + NADP --> CO2 + NADPH + PYR | Mae | b2463 |
| MAL + NAD --> CO2 + NADH + PYR | Sfc | b1479 |
| ICIT --> GLX + SUCC | AceA | b4015 |
| ACCOA + GLX --> COA + MAL | AceB | b4014 |
| ACCOA + GLX --> COA + MAL | GlcB | b2976 |
| PPI --> 2.00 PI | Ppa | b4226 |
| PPPI --> PI + PPI | PpxA | b2502 |
| PPI --> 2.00 PI | PpxB | b2502 |
| PPPI --> PI + PPI | SureEA | b2744 |
| PPI --> 2.00 PI | SureEB | b2744 |
| NADH + Q --> NAD + QH2 + 3 HEXT | Nuo | b2276,b2277,b2278,b2279,b2280,b2281,b2282,b2283,b2284,b2285,b2286,b2287,b2288 | Oxidative phosphorylation |
| NADH + Q --> NAD + QH2 | Ndh | b1109 |
| FOR + Q --> QH2 + CO2 + HEXT | Fdoec | b3892,b3893,b3894 |
| FOR + Q --> QH2 + CO2 + HEXT | Fdn | b1474,b1475,b1476 |
| GL3P + Q --> T3P2 + QH2 | GlpD | b3426 |
| GL3P + Q --> T3P2 + QH2 | GlpA | b2241,b2242,b2243 |
| QH2 + 0.5 O2 --> Q + 2.5 HEXT | CyoA | b0429,b0430,b0431,b0432 |
| PYR + NADH <--> NAD + LAC | Dld | b2133 |
| PYR + NADH <--> NAD + LAC | Ldh | b1380 |
| LAC + Q --> 1 PYR + 1 QH2 | Dld | b2133 |
| QH2 + 0.5 O2 --> Q + 2 HEXT | CbdAB | b0978,b0979 |
| NADPH + Q --> NADP + QH2 | MdaB | b0978,b0979 |
| PYR + Q --> AC + CO2 + QH2 | PoxB | b0871 |
| QH2 + 0.5 O2 --> Q + 4 HEXT | CydA | b0733,b0734 |
| NADPH + NAD --> NADP + NADH | Pnt | b1602,b1603 |
| NADP + NADH + 2 HEXT <--> NADPH + NAD | SthA | b3962 |
| ATP <--> ADP + PI + 4 HEXT | AtpF0, AtpF1, AtpI | b3736,b3737,b3738,b3731,b3732,b3733,b3734,b3735,b3739 |
| ATP --> ADP + PPPI | PpkA | b2501 |
| ATP + PI --> ADP + PPI | PpkB | b2501 |
| NADPH + NAD --> NADP + NADH | SthA | b3962 |
| SUCC + Q --> QH2 + FUM | Sdh | b0721,b0722,b0723,b0724 |
| O2xt <--> O2 | O2TXR |  |
| CO2xt <--> CO2 | CO2TXR |  |
| ATP --> ADP + PI | ATPM |  |
| LCTS --> GLC + bDGLAC | LacZ | b0344 | Alternate carbon metabolism and related reactions |
| LCTS --> GLC + bDGLAC | BglX | b2132 |
| bDGLAC <--> GLAC | GALM1R | b0756 |
| bDGLC <--> GLC | GALM2R | b0756 |
| GLAC + ATP <--> GAL1P + ADP | GalK | b0757 |
| GAL1P + UTP <--> PPI + UDPGAL | GalT | b0758 |
| GL + ATP --> ADP + GL3P | GlpK | b3926 |
| GL3P + NADP <--> T3P2 + NADPH | GpsA | b3608 |
| RIB + ATP --> R5P + ADP | RbsK | b3752 |
| UDPGAL <--> UDPG | GalE | b0759 |
| UTP + G1P <--> PPI + UDPG | GalUec | b1236 |
| G1P <--> G6P | Pgmec | b0688 |
| G1P <--> G6P | YqaB | b2690 |
| ATP + AMP --> 2 ADP | Adk | b0474 | Nucleotide Salvage Pathway |
| 41.25 ATP + 3.54 NAD + 18.22 NADPH + 0.20 G6P + 0.07 F6P + 0.89 R5P + 0.36 E4P + 0.12 T3P1 + 1.49 3PG + 0.51 PEP + 2.83 PYR + 3.74 ACCOA + 1.78 OA --> 3.74 COA + 1.07 AKG + 41.25 ADP + 41.250 PI + 3.54 NADH + 18.22 NADP + Biomass |  |  | Biomass reaction |
| Biomass + 41.25 ATP --> 41.25 ADP + 41.25 PI |  |  | Growth |
| Transport reactions | | |  |
| FORxt <--> FOR | FocA | b0904 | Formate transport via diffusion |
| LCTSxt + HEXT <--> LCTS | LacY | b0343 | Lactose transport via proton symport |
| FORxt <--> FOR | FocB | b2492 | Formate transport via diffusion |
| ETHxt + HEXT <--> ETH | ETHUPR |  |  |
| SUCCxt + HEXT <--> SUCC | DctA | b3528 | Succinate transport via proton symport 2 H |
| SUCCxt + HEXT <--> SUCC | DcuB | b4123 | Succintate transport via proton symport 3 H |
| SUCCxt + HEXT <--> SUCC | DcuA | b4138 | Succintate transport via proton symport 3 H |
| SUCC --> SUCCxt + HEXT | DcuC | b0621 | Succintate transport via proton symport 3 H |
| PYRxt + HEXT <--> PYR | PYRUPR |  |  |
| PIxt + HEXT <--> PI | PitA | b3493 | Phosphate reversible transport via symport |
| PIxt + HEXT <--> PI | PitBec | b2987 | Phosphate reversible transport via symport |
| GLCxt + HEXT --> GLC | GalP | b2943 | Glucose transport in via proton symport |
| G6Pxt + HEXT --> G6P | UhpT | b3666 | Glucose 6 phosphate transport via phosphate antiport |
| GLCxt + PEP --> G6P + PYR | Crr and PtsG and PtsH and PtsI | b2417,b1101,b2415,b2416 | Glucose transport via PEPPyr PTS |
| GLCxt + PEP --> G6P + PYR | Crr and MalX and PtsH and PtsI | b2417,b1621,b2415,b2416 | Glucose transport via PEPPyr PTS |
| GLCxt + PEP --> G6P + PYR | ManX and ManY and ManZ and PtsH and PtsI | b1817,b1818,b1819,b2415,b2416 | Glucose transport via PEPPyr PTS |
| GLxt --> GL | GlpF | b3927 | Glycerol transport via channel |
| RIBxt + ATP --> RIB + ADP + PI | RbsA and RbsB and RbsC and RbsDec | b3749+b3751+b3750 | Ribose transport via ABC system |
| ACxt + HEXT <--> AC | ACUPR | b4067 | Acetate reversible transport via proton symport |
| LAC <--> LACxt + HEXT | LldP | b3603 | Lactate reversible transport via proton symport |
| LAC <--> LACxt + HEXT | GlcA | b2975 | Lactate reversible transport via proton symport |
| GLCxt <--> |  |  | Glucose exchange |
| G6Pxt <--> |  |  | Glucose 6 phophate exchange |
| RIBxt <--> |  |  | Ribose exchange |
| GLxt <--> |  |  | Glycerol exchange |
| SUCCxt <--> |  |  | Succinate exchange |
| PYRxt <--> |  |  | Pyruvate exchange |
| LACxt <--> |  |  | Lactate exchange |
| LCTSxt <--> |  |  | Lactose exchange |
| FORxt <--> |  |  | Formate exchange |
| ETHxt <--> |  |  | Ethanol exchange |
| ACxt <--> |  |  | Acetate exchange |
| PIxt <--> |  |  | Phosphate exchange |
| CO2xt <--> |  |  | Carbon dioxide exchange |
| O2xt <--> |  |  | Oxygen exchange |

Note that reactions catalyzed by more than one protein/protein complex are listed multiple times. The data are based on information published in Covert et al. (Bioinformatics 24:2044, 2008) and the most recent reconstruction (denoted by iAF1260) of the *E. coli* metabolic network (see Feist et al., Mol Sys Biol 3:121, 2007; http://gcrg.ucsd.edu/In_Silico_Organisms/E_coli/E_coli_SBML). Metabolite names: 13PDG 1,3-bis-phosphoglycerate; 2PG 2-phosphoglycerate; 3PG 3-phosphoglycerate; AC acetate; ACCOA acetyl CoA; ACTP acetyl phosphate; ACxt acetate; ADP adenosine diphosphate; AKG alpha ketoglutaric acid; AMP adenosine monophosphate; ATP adenosine triphosphate; CIT citrate; CO2 carbon dioxide; CO2xt carbon dioxide, external; COA coenzyme A; D6PGC D-6-phosphogluconate; D6PGL D-6-phosphogluconolactone; E4P D-erythrose-4-phosphate; ETH ethanol; F6P fructose-6-phosphate; FDP fructose-1,6-biphosphate; FOR formic acid; FUM fumaric; G1P glucose-1-phosphate; G6P glucose-6-phosphate; G6Pxt glucose-6-phosphate, external; GAL1P galactose-1-phosphate; GL glycerol; GL3P glycerol-3-phosphate; GLAC galactose; GLC glucose; GLX glyoxylate; HEXT external H+; ICIT iso-citric acid; LAC D-lactate; LCTS Lactose; MAL maltose; NAD nicotinamide adenine dinucleotide; NADH nicotinamide adenine dinucleotide, reduced; NADP nicotinamide adenine dinucleotide phosphate; NADPH nicotinamide adenine dinucleotide phosphate, reduced; O2 oxygen; OA oxaloacetate; PEP phosphoenolpyruvate; PI phosphate; PPI pyrophosphoric acid; PPP inorganic triphosphate; PYR pyruvate; Q ubiquinone; QH2 ubiquinol; R5P ribose-5-phosphate; RIB ribose; RL5P ribulose-5-phosphate; S7P sedo heptulose; SUCC succinate; SUCCOA succinyl CoA; T3P1 glyceraldehyde-3-phosphate; T3P2 dihydroxyacetone phosphate; UDPG uridine diphosphate glucose; UDPGAL uridine diphosphate galactose; UTP uridine triphosphate; X5P xylulose-5-phosphate; bDGLAC beta-D-galactose; bDGLC beta-D-glucose. Additional abbreviations: ex exchange; xt extracellular.
