# Supplementary figures and images for: On the Accessibility of Adaptive Phenotypes of a Bacterial Metabolic Network
Source: PLoS Comput Biol. 2009 Aug 21;5(8):e1000472. doi: 10.1371/journal.pcbi.1000472 (PMC2716542; doi:10.1371/journal.pcbi.1000472)

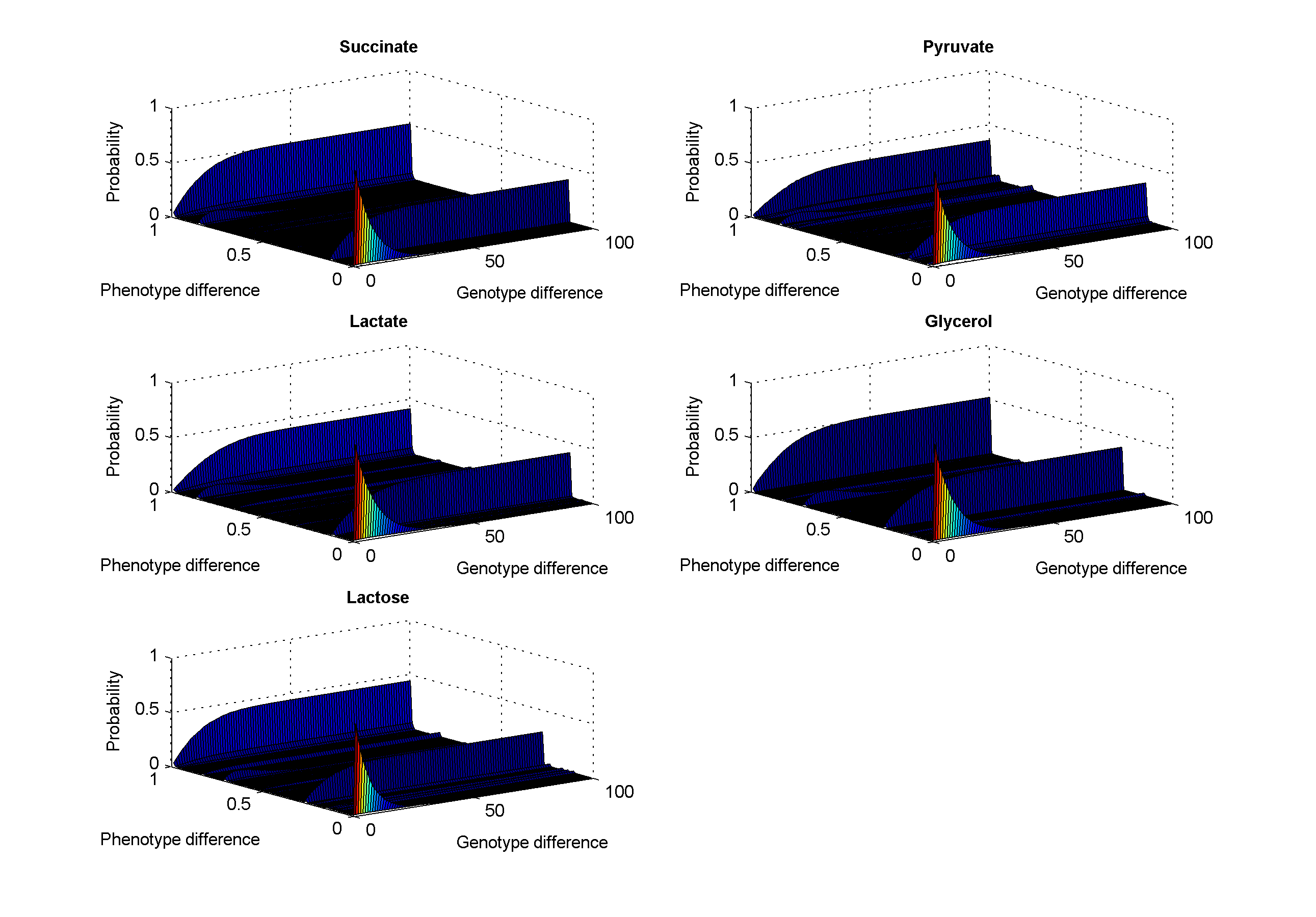

Supplement: Figure S1 — Conditional probability distribution of phenotype differences. The distributions were computed as described in the main text. Phenotype differences were binned using bins of sizes 0.01. (1.05 MB TIF) [file pcbi.1000472.s002.tif]

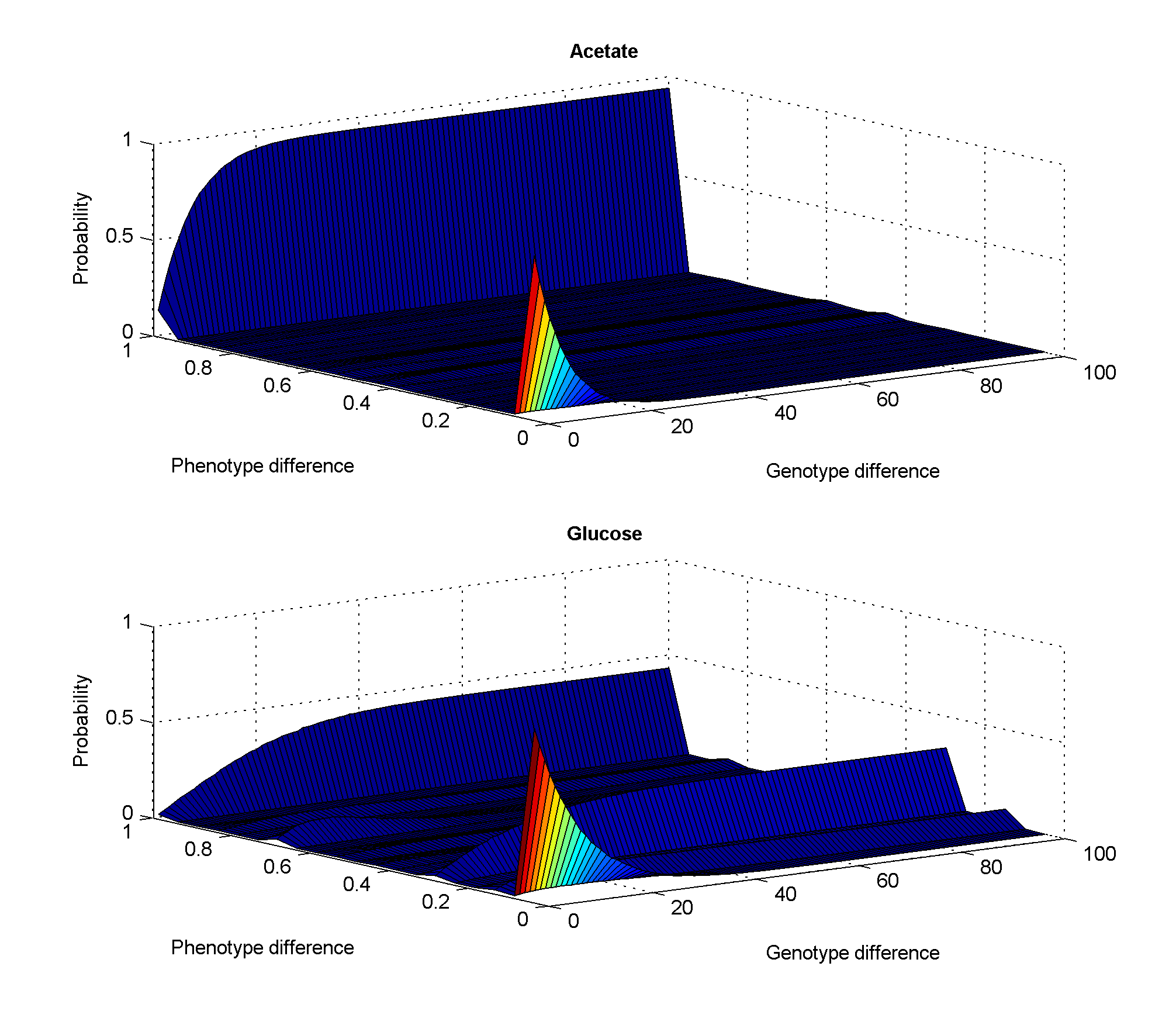

Supplement: Figure S2 — Conditional probability distribution of phenotype differences. The distributions were computed as described in the main text. Phenotype differences were binned using bins of sizes 0.05. (1.72 MB TIF) [file pcbi.1000472.s003.tif]

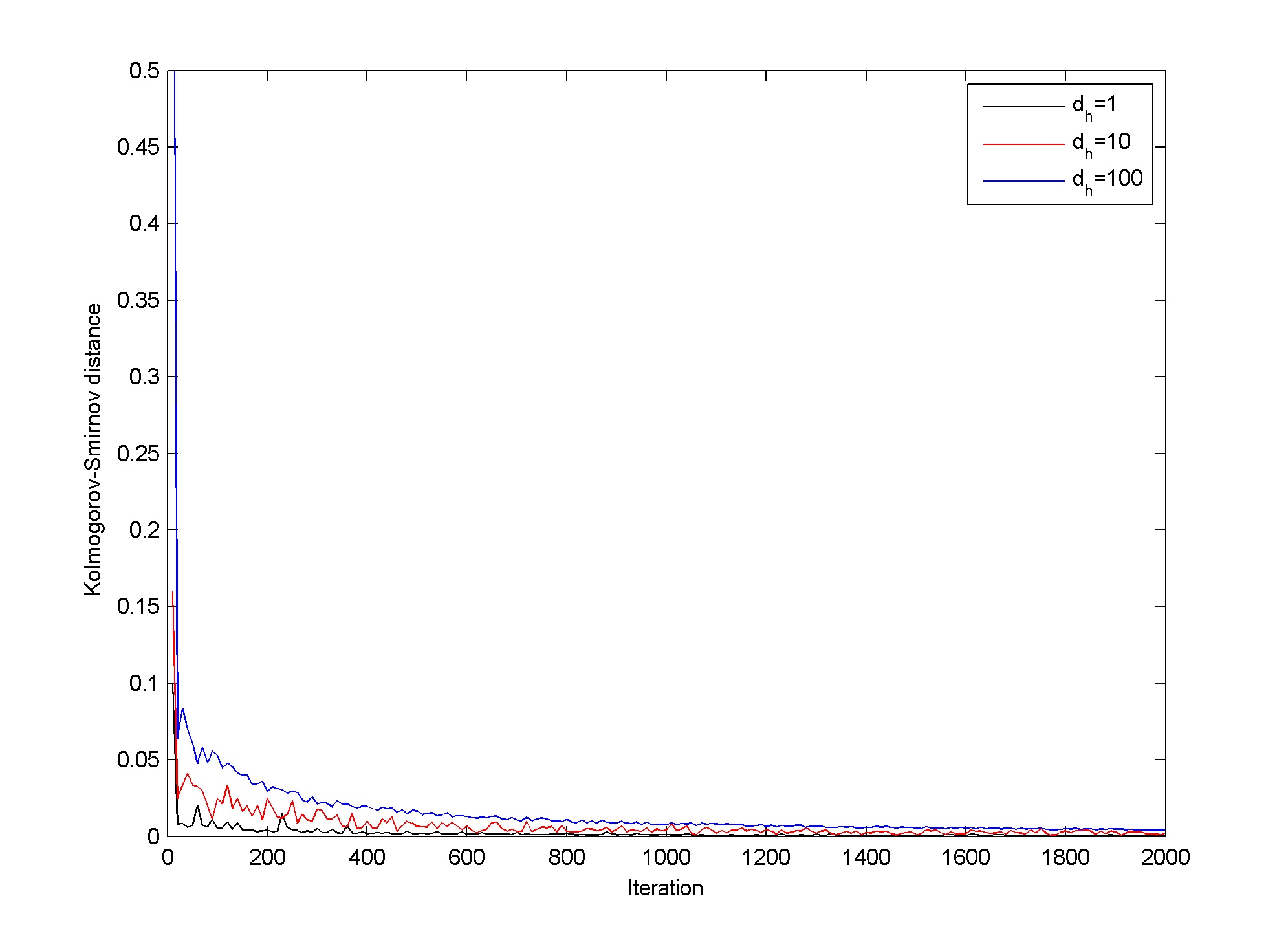

Supplement: Figure S3 — Convergence of the conditional probability distribution of phenotype differences. Shown is the Kolmogorov-Smirnov distance between the distribution of phenotype differences de conditioned on genotype differences dh obtained after t iterations of the uniform sampling algorithm described in the main text (denoted p(de|dh,t)) and the distribution p(de|dh,t-10), for three values of dh spanning a wide range. The Kolmogorov-Smirnov distance is given by max{abs(p(de|dh,t)-p(de|dh,t-10))}. The data were collected in a glucose environment. (0.21 MB TIF) [file pcbi.1000472.s004.tif]

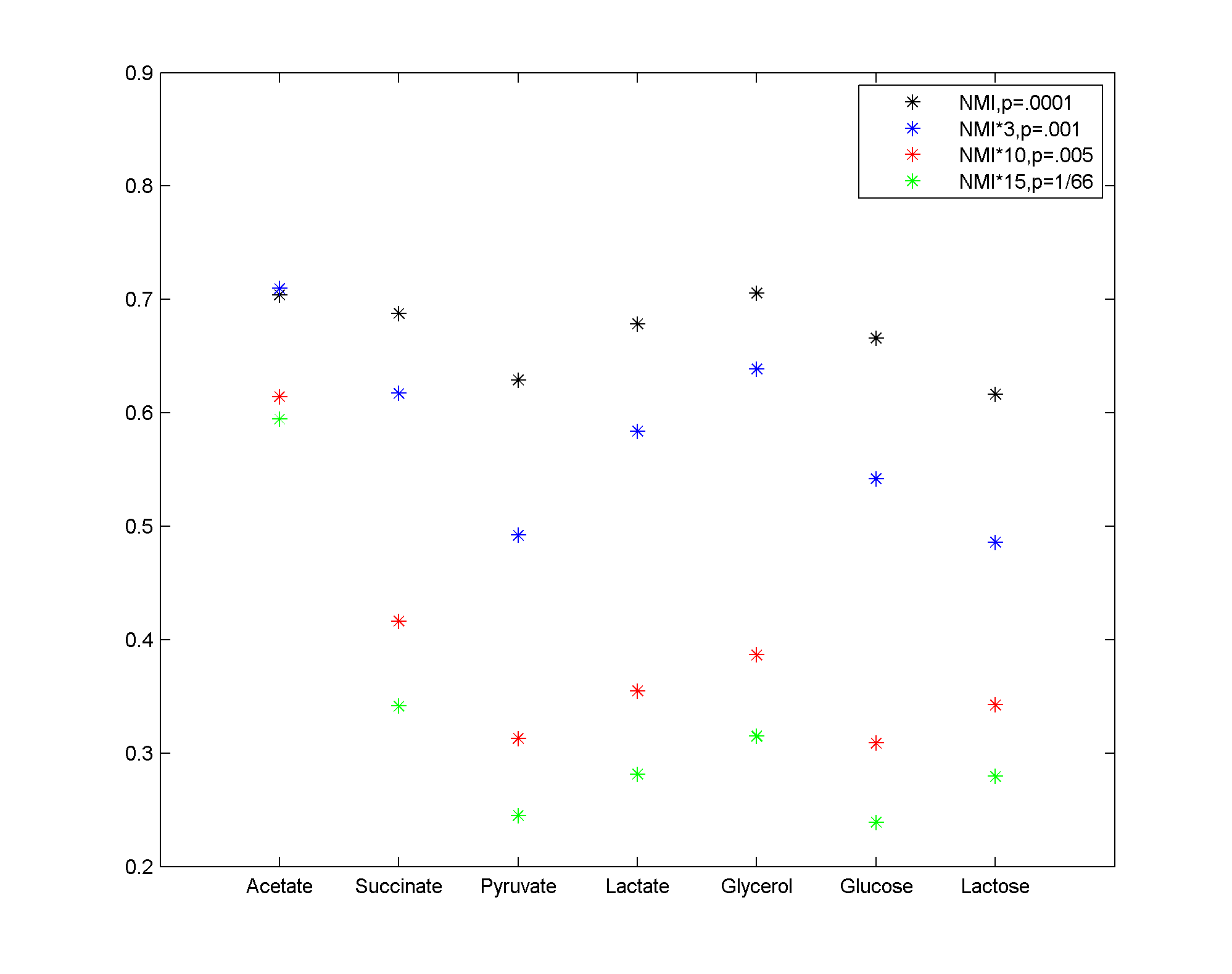

Supplement: Figure S4 — Rank-ordering of metabolic environments based on the normalized mutual information (NMI). The environments are listed in increasing order of quality, except in the case of lactose whose position in the rank-ordering is not known precisely. The NMI was computed as described in the main text, using different values of p, the mutation rate per genotype position. The measurement scales of NMI values corresponding to different values of p were adjusted in order to facilitate their presentation on the same graph. (0.20 MB TIF) [file pcbi.1000472.s005.tif]
